# Snm1B Interacts with PSF2

**DOI:** 10.1371/journal.pone.0049626

**Published:** 2012-11-26

**Authors:** Jay R. Stringer, Christopher M. Counter

**Affiliations:** Department of Pharmacology and Cancer Biology, Duke University Medical Center, Durham, North Carolina, United States of America; Tulane University Health Sciences Center, United States of America

## Abstract

The protein Snm1B plays a key role in interstrand crosslink (ICL) repair. In a yeast two-hybrid screen we identified the protein PSF2 to bind Snm1B. PSF2 is a member of the GINS complex involved in replication initiation and elongation, and is known to play a role in ICL repair. Snm1B was shown to bind PSF2 in human cells through two regions, strongly to a 144 amino acid N-terminal region and weakly to a second smaller 37 amino acid C-terminal region. Ectopic expression of PSF2 increased the amount of Mus81, a protein component of the endonucleolytic complex involved in ICL repair, co-immunoprecipitating with Snm1B. Moreover, deleting the N-terminal, but not C-terminal region of Snm1B reduced the amount of co-immunoprecipitated Mus81. Conversely, the telomere-binding protein TRF2 competed with PSF2 for binding to the C-terminus of Snm1B, and deletion of this region, but not the N-terminal region, reduced Snm1B chromatin association. We speculate that the N-terminal region of Snm1B forms a complex containing PSF2 and Mus81, while the C-terminal region is important for PSF2-mediated chromatin association.

## Introduction

Interstrand crosslinks (ICLs) are toxic lesions that covalently attach opposite strands of DNA [1]. One protein involved in the repair of ICLs is Snm1B (Apollo/Dclre1B) [2], [3], [4], [5], [6], [7]. Snm1B is a 60 kDa protein belonging to the β-CASP family of proteins, which also contains Snm1A (Dclre1A) and Snm1C (Artemis/Dclre1C) [8]. These proteins are characterized by β-CASP and Metallo-β-Lactamase domains responsible for nucleic acid hydrolysis [8], and all three proteins have inherent 5′-3′ DNA exonuclease activity [6], [9], [10], [11]. In mammalian and chicken cells, Snm1A plays a role in ICL repair, likely in a different repair pathway than Snm1B [12], [13], [14]. Snm1C is involved in nonhomologous end joining and has a structure-specific endonuclease activity dependent upon binding DNA-PKcs [9], [15]. Snm1B is required for proper ICL repair, as knockdown of this protein leads to sensitivity of cells to ICLs [2], [3], [4], [13] and blocks the formation of double-strand breaks (DSBs) that occur as an intermediate in ICL repair [3], [16]. The enzymatic activity of Snm1B appears to be dispensable for ICL repair, although the conserved β-CASP and Metallo-β-Lactamase domains are required [3]. Interestingly, Snm1B associates with Mus81 through the Metallo-β-Lactamase domain [3]. Mus81 and Eme1 form the structure-specific endonuclease complex Mus81/Eme1 [17], [18] that is important for cleavage of replication fork substrates *in vitro* [17], [18], and for the formation of DSBs after ICL formation during replication *in vivo* [19]. These results suggest that association of Snm1B with Mus81 is important for ICL repair during DNA replication [3].

In addition to the role of Snm1B in ICL repair, Snm1B protects replicating telomeres from being detected as DNA damage through a C-terminal interaction with the telomere-binding protein TRF2 [5], [6], [7], [20]. Moreover, knockdown of Smn1B leads to an accumulation of DNA repair proteins on telomeres [7], indicative of telomere dysfunction [21]. The telomere protection and ICL repair functions of Snm1B appear to be separate from one another. Specifically, a patient with a rare form of dyskeratosis congenital termed Hoyeraal–Hreidarsson syndrome, was reported to encode a truncated form of Snm1B that failed to bind TRF2 and exhibited telomere DNA damage, but was not sensitive to ICL-inducing agents [22].

The above observations suggest protein-protein interactions may mediate the different functions of Snm1B. We screened a yeast two-hybrid library for novel protein interactions using the very C-terminus of Snm1B as a bait. This region was already known to interact with at least one protein, TRF2 [5], [6], [7], and moreover, a C-terminal deletion mutant was both poorly expressed and reduced the viability of cells, at least in ectopic over-expression settings [23], suggesting a functional contribution. The two-hybrid screen revealed an interaction with the protein PSF2. PSF2 is a 21 kDa protein that is one of four proteins that form the GINS complex. The GINS complex is a highly conserved protein complex [24] required for DNA replication and initiation through interacting with Cdc45 [25], [26], [27]. Of interest with regards to Snm1B, knockdown of PSF2 was recently shown to sensitize cells to ICLs [28]. We thus investigated whether PSF2 was a bona fide interacting protein of Snm1B, and the impact of deleting the regions responsible for this binding in Snm1B on TRF2 and Mus81 associations and chromatin loading.

**Figure 1:**
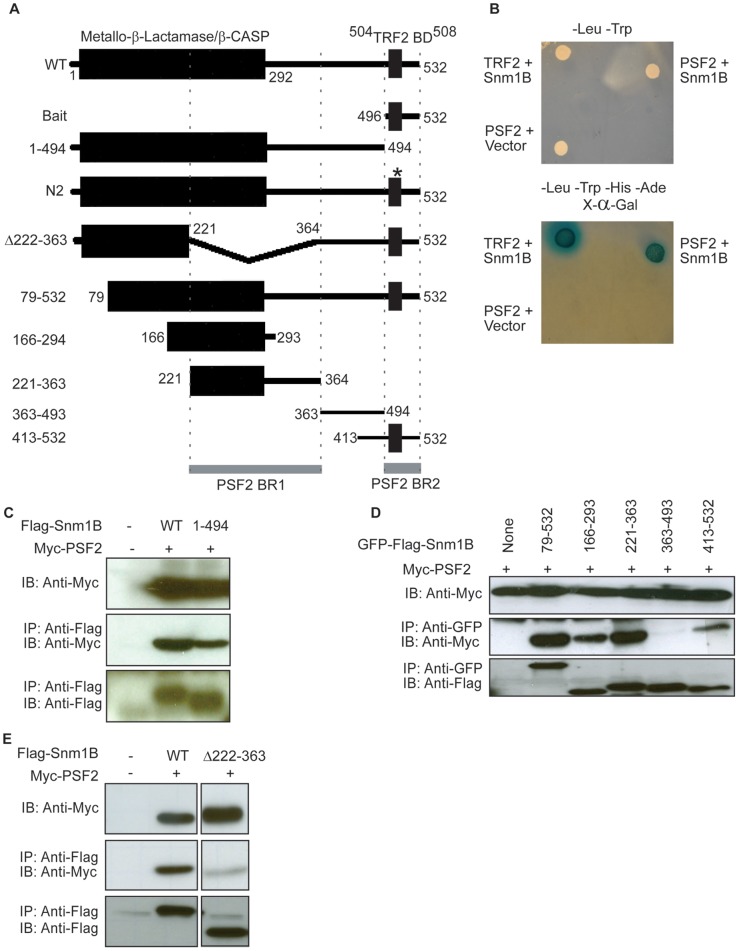
Snm1B interacts with PSF2. *A,* Diagram of Snm1B mutants generated. The two putative PSF2-binding regions (PSF2 BR1 and BR2) are denoted by grey bars. WT, wild type. * denotes a mutation that reduces TRF2 binding. *B,* Growth of yeast expressing PSF2-Gal4AD (PSF2) and the Gal4BD (vector), PSF2-GalAD and Gal4BD fused to the C-terminus of Snm1B (Snm1B), or positive control TRF2-Gal4AD (TRF2) and Snm1B-GalBD on SD/-leu/-trp or SD/-ade/-his/-leu/-trp dropout media in the presence of x-α-gal. *C, E,* The indicated Flag epitope-tagged Snm1B (Flag-Snm1B) or *D,* the indicated GFP-tagged Flag-Snm1B (GFP-Flag-Snm1B) transiently expressed in 293T cells in the presence of ectopic Myc epitope-tagged PSF2 (Myc-PSF2) were immunoprecipitated (IP) with either an anti-Flag or anti-GFP antibody and immunoblotted (IB) with an anti-Flag antibody to visualize Flag-Snm1B or an anti-Myc antibody to visualize Myc-PSF2. Data are representative of at least two independent experiments.

## Materials and Methods

### Plasmids

All Snm1B expression constructs were derived from PCR amplification of a Flag-Snm1B cDNA [5] and sequenced to confirm correct. The yeast two-hybrid Snm1B bait vector was created by cloning two tandem in-frame copies of the sequence encoding the last 37 amino acids of Snm1B into pGBKT7 (Clonetech). Previously described Flag-Snm1B-WT, Flag-Snm1B^1–494^, and Flag-Snm1B^N2^ cDNAs [23] were PCR amplified and subcloned into pLPC (a gift from Susan Smith). Flag-Snm1B^Δ222–363^ was created by two separate PCR amplifications to generate regions spanning amino acids 1 to 221 and 364 to 532, which were then simultaneously ligated into pLPC, resulting in the described internal deletion having the addition of the sequence 5′-CGCGGCCGC-3′ (a NotI restriction site and one base pair to keep in frame) between amino acids 221 and 364. Flag-Snm1B^79–532^ and Flag-Snm1B^413–532^ were cloned by PCR amplification to include a 5′ Flag epitope tag and sequence corresponding to the indicated amino acids, and then subcloned into pEGFP-C3 (Clonetech). Flag-Snm1B^166–293^, Flag-Snm1B^221–363^, and Flag-Snm1B^363–494^ were created by first inserting a stop codon at amino acids 294, 364, and 495 of Flag-Snm1B by site directed mutagenesis followed by PCR amplification with a 5′ primer designed to create a Flag epitope-tag at the indicated amino acids of the construct and a 3′ primer corresponding to the original 3′ of Flag-Snm1B, and then subcloned into pEGFP-C3. PSF2 was PCR amplified from clone MGC-673 (ATCC) and cloned into pCMV-MYC (Clontech). The F120A mutation was introduced into pcDNA3-myc-TRF2 [5] by site-directed mutagenesis. HA-MUS81 was created by PCR amplification of clone MGC:14953 (Imagenes) with primers that included a 5′ HA epitope tag and cloned into pcDNA3 (Invitrogen).

**Figure 2:**
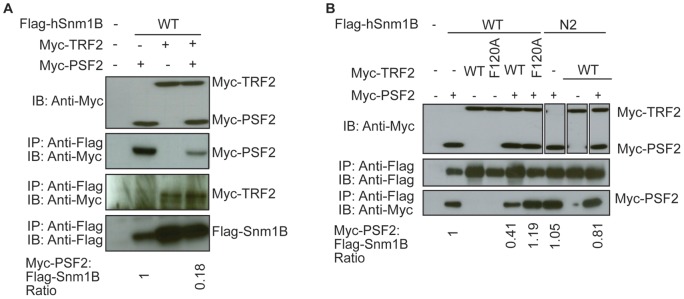
Myc-TRF2 disrupts the interaction between Flag-Snm1B and Myc-PSF2. *A, B,* The indicated wild type (WT) or N2 mutant Flag epitope-tagged Snm1B (Flag-Snm1B) and Myc epitope-tagged PSF2 (Myc-PSF2) proteins transiently expressed in 293T cells in the absence or presence of wild type (WT) or the F120A mutant of Myc epitope-tagged TRF2 (Myc-TRF2) were immunoprecipitated (IP) with anti-Flag antibody and immunoblotted (IB) with either an anti-Flag antibody to visualize Flag-Snm1B or an anti-Myc antibody to visualize Myc-PSF2 or Myc-TRF2. Bottom: normalized Myc-PSF2:Flag-Snm1B ratio. Data are representative of at least two independent experiments.

### Yeast Two-Hybrid Assay

Yeast strain AH109 expressing pGBKT7 encoding the bait protein comprised of the aforementioned Snm1B C-terminal region fused in frame to the Gal4 DNA-binding domain (Gal4BD) was used to screen the Matchmaker^TM^ Pretransformed Human Hela Library, according to the manufacture's protocol (Clonetech). Y187 yeast were then transformed with the prey vector pGADT7 encoding either PSF2 or TRF2 (identified in this screen) fused in frame with the Gal4 activation domain (Gal4AD) and mated with AH109 yeast containing either empty pGBKT7 or pGBKT7 expressing the bait and analyzed for growth on SD/-leu/-trp, as a control, and SD/-ade/-his/-leu/-trp drop-out plates supplemented with X-α-gal (Sigma) to show the interaction of the indicated proteins.

**Figure 3:**
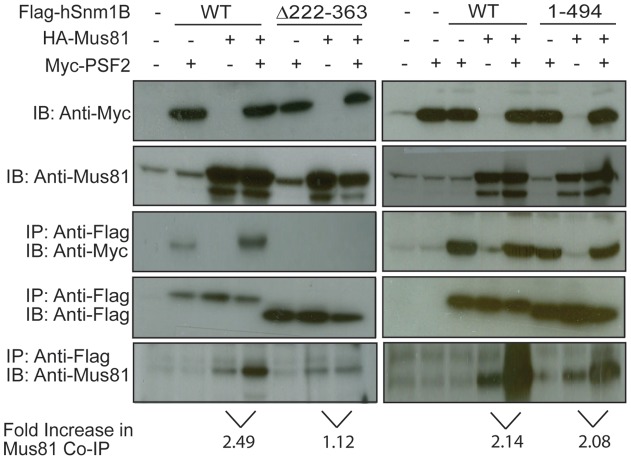
Flag-Snm1B co-immunoprecipitates with Mus81 and Myc-PSF2. The indicated wild type (WT) or mutant (Δ222–363 or 1–494) Flag epitope-tagged Snm1B (Flag-Snm1B) and HA epitope-tagged Mus81 (HA-Mus81) proteins transiently expressed in 293T cells in the absence or presence of Myc epitope-tagged PSF2 (Myc-PSF2) were immunoprecipitated (IP) with anti-Flag antibody and immunoblotted (IB) with an anti-Flag antibody to visualize Flag-Snm1B (WT, Δ222–363, or 1–494), anti-Myc antibody to visualize Myc-PSF2, or an anti-Mus81 antibody to visualize Mus81. Bottom: fold increase in Mus81 co-immunoprecipitating with Flag-Snm1B. Data are representative of at least two independent experiments.

### Transient Transfection

293T and Hela cells were transiently transfected with Fugene 6 (Roche) at approximately 40%–60% confluency according to the manufacturer's protocol. The cells were collected 36 to 48 hours later for analysis.

**Figure 4:**
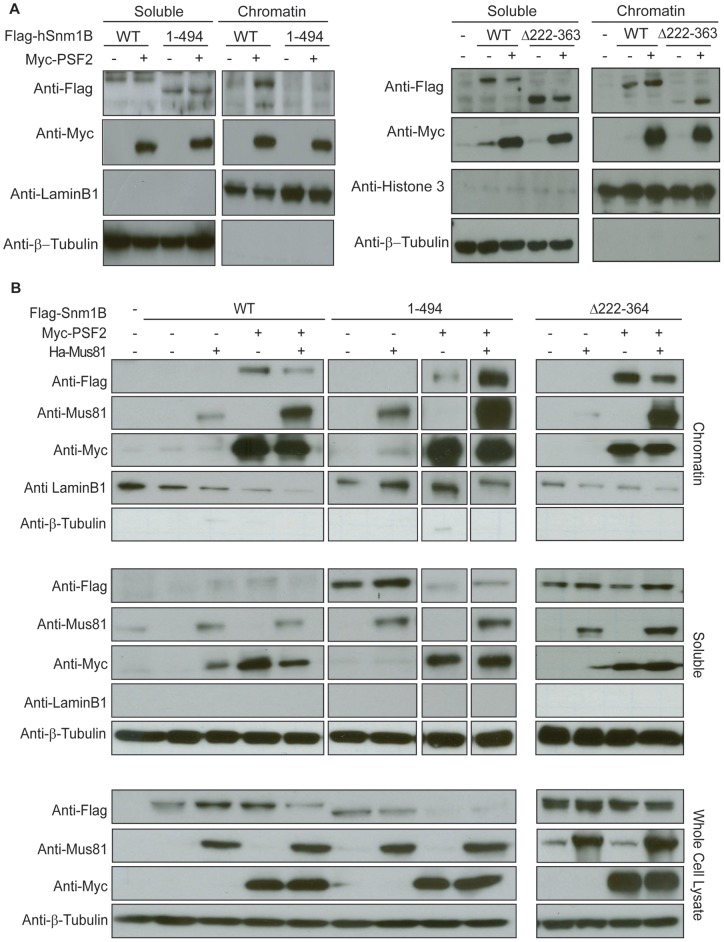
Flag-Snm1B is enriched in the chromatin fraction in the presence of Myc-PSF2. *A, B,* The indicated wild type (WT) or mutant (Δ222–363 or 1–494) Flag epitope-tagged Snm1B (Flag-Snm1B) proteins were transiently expressed in 293T cells alone or in the presence of either Myc epitope-tagged PSF2 (Myc-PSF2), HA epitope-tagged Mus81 (HA-Mus81), or both. Soluble, chromatin (insoluble), and whole cell lysates were immunoblotted with anti-Flag, anti-Myc, anti-Mus81, anti-β-tubulin, anti-lamin B1 or anti-histone H3 antibodies to visualize Flag-Snm1B (WT, Δ222–363, or 1–494), Myc-PSF2, Mus81, β-tubulin, lamin B1 or histone H3 proteins, respectively. Immunoblot exposures were optimized for each antibody, although the soluble and chromatin fractions immunoblotted for tubulin, histone H3, or lamin B1 were exposed for the same time. Exposure times for lanes 10–13 in *B* were different from lanes 1–9, as these samples were resolved on a different gel. Data are representative of at least two independent experiments.

### Co-Immunoprecipitation Analysis

The indicated transiently transfected 293T cells were lysed with RIPA buffer (50 mM Tris pH 8.0, 150 mM NaCl, 0.1% SDS, 0.5% sodium deoxycholate, 1% NP-40 supplemented with 1mM PMSF, 2 µg/mL aprotinin, and 1 µg/mL leupeptin) by homogenization through a 25G needle. Lysates were rotated at 4°C for 5–10 minutes and spun at 16,000*g* to remove debris and the protein concentration was determined by DC^TM^ Protein Assay (Bio-Rad). To immunoprecipitate Flag-tagged Snm1B protein, an equal amount of lysate (1–2 mg depending on experiment) was incubated with 15 µl of M2 affinity gel (Sigma) and rotated at 4°C overnight. To immunoprecipitate GFP-tagged (Flag-)Snm1B protein, an equal amount of lysate (1–2 mg depending on experiment) was incubated with 4 µg of anti-GFP antibody (Roche), rotated at 4°C overnight, after which 10 µl of GammaBind G Sepharose (GE/Amersham) was added and samples were rotated an additional hour. To immunoprecipitate Myc-tagged PSF2 protein, an equal amount of lysate (1–2 mg depending on experiment) was incubated with 4 µg of anti-Myc antibody (Invitrogen), rotated at 4°C overnight, after which 10 µl of GammaBind G Sepharose (GE/Amersham) was added and samples were rotated an additional hour. Immunoprecipitates were then washed twice with RIPA buffer, resolved by polyacrylamide gel electrophoresis, and immunoblotted with mouse anti-Flag M2 (1:1000 Sigma), mouse anti-Myc (1:5000 Invitrogen) or mouse anti-Mus81 (1:1000, Abcam) antibodies to detect Flag-tagged Snm1B, GFP-tagged Flag-Snm1B, Myc- tagged PSF2 and TRF2, or Mus81 proteins, respectively. Exposures were optimized to detect the different proteins, and hence vary between different antibodies.

### Chromatin Fractionation and Immunoblot Analysis

The chromatin and soluble fractions of the indicated transiently transfected 293T cells were isolated with 0.1% Triton X-100 CSK buffer (10 mM PIPES pH 6.8, 100 mM NaCl, 300 mM sucrose, 3 mM MgCl_2_, 1 mM EGTA, 1 mM EDTA, 50 mM NaF, 5 mM Na3VO4 supplemented with the addition 1 mM PMSF, 2 µg/mL aprotinin, and 1 µg/mL leupeptin) as previously described [28]. The soluble fraction was centrifuged at 16,000*g* to remove remaining debris. The insoluble pellet was resuspended in 1x laemmli buffer (60mM Tris-Cl pH 6.8, 2% SDS, 5% glycerol, 100 mM dithiothreitol, and 0.01% bromophenol blue) and sonicated for 30 seconds at 25% amplitude on a Fisher Scientific sonic dismembrator model 150. Whole cell lysates were isolated with RIPA buffer as described above. 50 µg of protein from the soluble, chromatin, and whole cell lysates were resolved by SDS polyacrylamide gel electrophoresis and immunoblotted as above with anti-Flag M2, mouse anti-Myc, mouse anti-Mus81, anti-lamin B1 (1:1000, Invitrogen), anti-histone H3 (1:1000, Cell Signaling) and anti-β-tubulin (1:1000, Sigma) antibodies to detect Flag-Snm1B, Myc-PSF2, Mus81, the nuclear protein laminin B1, the chromatin protein histone H3, and the cytosolic protein β-tubulin, respectively. Exposures were optimized to detect the different proteins, and hence vary between different antibodies.

### ImageJ Quantification

Quantification of immunoblots was performed with ImageJ [29]. The ratio of co-immunoprecipitated Myc-PSF2 to Flag-Snm1B was determined by quantifying the densitometry signals of Myc-PSF2 and Flag-Snm1B immunoblot, with the Myc-PSF2/Flag-Snm1B (WT) ratio normalized to one. The fold increase in Mus81 co-immunoprecipitation was calculated for each Flag-Snm1B protein (WT, 1–495, or Δ222–363) by dividing the value of the co-immunoprecipitated Mus81 when Myc-PSF2 is expressed by the value of the co-immunoprecipitated Mus81 when Myc-PSF2 is not expressed.

## Results

### A Yeast Two-Hybrid Screen Identified an Interaction Between Snm1B and PSF2

The very C-terminus of Snm1B may serve a regulatory role [23] and was already known to bind to at least one protein, the telomere-binding protein TRF2 [5], [6], [7], [20]. Thus, we used a tandem repeat of the last 37 amino acids of human Snm1B as bait (Figure. 1a) to screen a human cDNA prey library by the yeast two-hybrid methodology for potential protein interactions. A total of 134 clones were sequenced corresponding to 8 unknown sequences and 35 annotated genes, including the aforementioned TRF2 (Table. S1). One of these potential interacting proteins was the PSF2 subunit of the GINS complex, which was of particular interest given that PSF2, like Snm1B, is required for ICL repair [28], [30]. We confirmed that the PSF2 prey vector required the Snm1B bait vector to activate the Ade, His, and Mel1 reporter genes, as indicated by blue colony growth of the reporter strain of yeast on the appropriate dropout media supplemented with X-α-gal (Figure. 1B). This growth was less than that observed with the positive control TRF2 prey vector, suggesting a weak interaction. We conclude that the C-terminus of Snm1B binds, likely directly, to the GINS subunit PSF2.

### Two Regions of Snm1B Mediate Co-Immunoprecipitation with PSF2 in Human Cells

To evaluate whether full-length Snm1B binds to full-length PSF2 in human cells, human 293T cells were co-transfected with expression plasmids encoding N-terminal Flag epitope-tagged human Snm1B (Flag-Snm1B) and N-terminal Myc epitope-tagged human PSF2 (Myc-PSF2). The expressed Flag-Snm1B protein was then immunoprecipitated by virtue of the Flag tag from these cells and immunoblotted with an anti-Myc antibody to detect Myc-PSF2, revealing that the two proteins co-immunoprecipitated (Figure. 1C). The reciprocal was also true, namely that Flag-Snm1B co-immunoprecipitated with Myc-PSF2, although this only occurred in the presence of HA-Mus81 (see below, Figure. S1A, B). Interestingly, when the experiment was performed with Flag-Snm1B^1–494^, a mutant of Flag-Snm1B lacking the portion of Snm1B used in the two-hybrid screen (Figure. 1A), Snm1B still co-immunoprecipitated with Myc-PSF2, although at a reduced efficiency (Figure. 1C). These results suggest the presence of another PSF2-binding region within Snm1B.

To map the putative second PSF2-binding region, a series of N- and C-terminal truncation mutants of Flag-Snm1B were generated and fused in frame to GFP to increase the size of small fragments to facilitate expression and detection. These proteins were co-expressed in 293T cells with Myc-PSF2, after which protein association was determined by immunoprecipitating the GFP-Flag-Snm1B mutants followed by immunoblot to detect Myc-PSF2. This analysis revealed that any fragment of GFP-Flag-Snm1B containing the region spanning amino acids 221 to 363 co-immunoprecipitated with Myc-PSF2 (Figure. 1D). Indeed, the minimal GFP-Flag-Snm1B^221–363^ fragment encoding only amino acids 221 to 363 co-immunoprecipitated with roughly three-fold more Myc-PSF2 than GFP-Flag-Snm1B^413–532^ fragment that contains the bait region (Figure. 1D). Conversely, Flag-Snm1B^Δ222–363^, in which this second region was deleted, co-immunoprecipitated with approximately six-fold less Myc-PSF2 compared to Flag-Snm1B^WT^ (Figure. 1E). We suggest that two regions of Snm1B encompassed by amino acids 221–363 and 496–532 are responsible for the association with PSF2, with the N-terminal region accounting for most of the affinity of Snm1B for PSF2 (Figure. 1A).

### TRF2 Reduces the Amount of PSF2 Co-Immunoprecipitating with Snm1B

Given that TRF2 binds to one of the regions of Snm1B associating with PSF2 [5], we evaluated the impact of TRF2 expression on the newly identified interaction of Snm1B with PSF2. Specifically, 293T cells, either untransfected or co-transfected with expression plasmids encoding Flag-Snm1B, Myc-PSF2, and/or Myc-TRF2, were lysed after which Flag-Snm1B was immunoprecipitated and immunoblotted with anti-Myc or anti-Flag antibodies to detect Flag-Snm1B, Myc-PSF2, and/or Myc-TRF2. As expected, Flag-Snm1B co-immunoprecipitated with Myc-TRF2 [5], [6], [7] and Myc-PSF2. However, when both these proteins were co-expressed with Flag-Snm1B, the amount of Myc-PSF2, but not Myc-TRF2, that co-immunoprecipitated with Flag-Snm1B was reduced (Figure. 2A). When the level of immunoprecipitated Myc-PSF2 was normalized to the level of Flag-Snm1B, to account for the increase in Flag-Snm1B when co-expressed with Myc-TRF2 [23], ectopic Myc-TRF2 reduced the amount of Myc-PSF2 co-immunoprecipitating with Flag-Snm1B by more than half (Figure. 2). This result was validated by mutational analysis. Specifically, we demonstrate that Myc-TRF2^F120A^, a mutant of TRF2 that cannot bind Snm1B [31], failed to reduce the amount of Myc-PSF2 that co-immunoprecipitated with Flag-Snm1B. Similarly, the N2 mutant of Flag-Snm1B that has reduced binding to TRF2 ([23], [31] and data not shown) readily co-immunoprecipitated with Myc-PSF2 in the presence of wild type Myc-TRF2 (Figure. 2B) [23], [31]. We therefore suggest that TRF2 can inhibit the association of Snm1B with PSF2.

### PSF2 Increases the Amount of Mus81 Co-Immunoprecipitating with Snm1B

The Mus81 subunit of the structure specific endonuclease complex Mus81/Eme1 is required for the formation of DSBs after ICL formation during replication *in vivo*, and binds to Snm1B [3], [19]. Given these observations and that PSF2, as part of the GINS complex, is a member of the replication fork [32], we measured the impact of PSF2 expression on the association of Snm1B with Mus81. Specifically, 293T cells, either untransfected or transiently co-transfected with expression plasmids encoding Flag-Snm1B, Myc-PSF2, and/or HA-Mus81, were lysed, after which Flag-Snm1B was immunoprecipitated and immunoblotted to detect Flag-Snm1B, Myc-PSF2, or Mus81. As previously reported, Flag-Snm1B co-immunoprecipitated with Mus81 [3]. However, the addition of Myc-PSF2 increased this association by more than two fold (Figure. 3). To determine which of the two PSF2-binding regions was responsible for this increase, the experiment was repeated with Flag-Snm1B^Δ222–363^, which lacks the first PSF2-binding region accounting for most of the association with PSF2, and Flag-Snm1B^1–494^, which lacks the second weaker PSF2-binding region (Figure. 1A, 1C, 1E). The ability to co-immunoprecipitate more Mus81 in the presence of Myc-PSF2 was significantly reduced in Flag-Snm1B^Δ222–363^, but not Flag-Snm1B^1–494^ (Figure. 3), although Myc-PSF2 could also co-immunoprecipitate with HA-Mus81 in the absence of Flag-Snm1B (Figure. S1B). Admittedly, the reduction in HA-Mus81 association with Flag-Snm1B^Δ222–363^ in the presence of Myc-PSF2 could be due to reduced Mus81 binding to Snm1B, although the Mus81-binding site maps to amino acids 68–139 in Snm1B [3]. Additionally, while Myc-PSF2 co-immunoprecipitated with Flag-Snm1B, the reverse co-immunoprecipitation required ectopic HA-Mus81 (Figure. S1A, B). Taken together, we suggest that interactions between Snm1B, PSF2, and Mus81 promote the formation of a larger complex, with Snm1B binding to PSF2 through the N-terminal region.

### Snm1B is Enriched in the Chromatin Fraction through the C-Terminal Interaction with PSF2

Since PSF2 accumulates in chromatin through forming a complex with Cdc45 and MCM2-7 [26], [33] and associates with Snm1B (Figures. 1, 2, and 3), we tested whether PSF2 localized Snm1B onto chromatin. 293T cells were transiently transfected with an expression vector encoding Flag-Snm1B or co-transfected with expression vectors encoding Flag-Snm1B and Myc-PSF2. Chromatin and soluble fractions were isolated and their purity validated by the appropriate absence or presence of the nuclear protein laminin B1 or the chromatin protein histone H3 and the cytosolic protein tubulin. Both fractions were then immunoblotted to detect Flag-Snm1B and Myc-PSF2, revealing that the amount of Flag-Snm1B in the chromatin fraction was increased upon co-expressing Myc-PSF2 (Figure. 4A).

To determine which PSF2-binding region was responsible for this enrichment, the experiment was repeated with Flag-Snm1B^Δ222–363^ and Flag-Snm1B^1–494^, which lack the N- and C-terminal PSF2-binding regions, respectively. This analysis revealed that the C-terminal PSF2-binding region was responsible for this enrichment. Specifically, while the levels of both wild type and Δ222–363 mutant Flag-Snm1B proteins in the chromatin fraction were increased in the presence of Myc-PSF2, very little Flag-Snm1B^1–494^ was detected in the chromatin fraction in the presence of Myc-PSF2 (Figure. 4A). We suggest that PSF2 promotes recruitment of Snm1B to chromatin through the C-terminal PSF2-binding region.

We then tested if the inability of Flag-Snm1B^1–494^ to associate with chromatin could be overcome by the expression of HA-Mus81. 293T cells were transiently co-transfected with expression vectors encoding the wild type or the 1–494 mutant versions of Flag-Snm1B with no transgene, Myc-PSF2, and/or HA-Mus81 (Figure. 4B). As already noted, very little Flag-Snm1B^1–494^ was detected in the chromatin fraction, with most of the protein remaining in the soluble fraction, even in the presence of Myc-PSF2 or HA-Mus81. However, in cells expressing both Myc-PSF2 and HA-Mus81, Flag-Snm1B^1–494^ was enriched in the chromatin fraction (Figure. 4B lane 9). This recruitment to chromatin is likely dependent upon the N-terminal PSF2-binding region of Snm1B. Specifically, when the experiment was repeated with Flag-Snm1B or Flag-Snm1B^Δ222–363^, each protein was still enriched in the chromatin fraction upon expression of Myc-PSF2, but this was not enhanced in the presence of HA-Mus81 (Figure. 4B). Thus, we suggest that Snm1B can be recruited to chromatin through the C-terminus directly by PSF2 and the N-terminal PSF2-binding region in a complex with PSF2 and Mus81.

## Discussion

We now report that PSF2 binds to Snm1B in the yeast two-hybrid assay and co-immunoprecipitates with Snm1B in human cells, indicating that PSF2 associates with Snm1B, probably directly. Mapping revealed that PSF2 bound to two distinct regions in Snm1B; strongly to an N-terminal region encompassed by amino acids 221 to 363 and weakly to a C-terminal region encompassed by amino acids 496 to 532. Interestingly, these two interaction regions impacted Snm1B functions differently. The C-terminal region competed with TRF2 binding and was required for chromatin association in the presence of PSF2, whereas the N-terminal region promoted an association with Mus81. We thus suggest that the interaction with PSF2 is related to Snm1B function.

The N-terminal region of Snm1B promoted complex formation between PSF2 and Mus81, which led to the accumulation of Snm1B on chromatin in the presence of both Mus81 and PSF2. Given the role of Mus81 in the cleavage step of ICL repair, perhaps the N-terminus fosters this step. Admittedly, assigning different roles to the N- and C-terminal PSF2-binding regions is likely an oversimplification, as both the Flag-Snm1B^Δ222–363^ and Flag-Snm1B^1–495^ mutants may alter more than PSF2 binding. Nevertheless, ectopic expression of PSF2 promoted the association of Snm1B with Mus81 and chromatin, independently supporting a role for these PSF2-binding regions in different functions of Snm1B. Another caveat to these interpretations is that Snm1B was over-expressed due to the inability to detect endogenous Snm1B [2], [6]. Nevertheless, indirect evidence corroborates the suggestion that the interaction with PSF2 has an impact on Snm1B function. Knockdown of either Snm1B or PSF2 sensitizes cells to ICLs [2], [3], [28]. Moreover, we speculate that both proteins are likely to function in the Fanconi Anemia pathway as knockdown of Snm1B does not increase the sensitivity of FANCD2-deficient cells to MMC, and PSF2 interacts with the FANCF protein [4], [28]. Given that PSF2 localizes to the replication fork, we further speculate that perhaps this protein recruits Snm1B to forks stalled at ICLs in chromatin. However, we cannot rule out that Snm1B could be associated with replication forks before they stall, as FANCA, FANCB, and FANCF are thought to associate with the replication fork independent of a DNA damage response through their interaction with PSF2 [28]. Finally, how ectopic PSF2 induces Snm1B into chromatin without overexpression of the other members of the GINS complex is uncertain, as PSF2 is not known to associate with chromatin separate from the GINS complex. As knockdown of one member of the GINS complex reduces the protein levels of the other members [27], perhaps overexpression of PSF2 may increase the levels of the other GINS proteins. In conclusion, we identified an interesting interaction linking Snm1B, a member of the ICL repair pathway, with PSF2, a component of the DNA replication fork.

## Supporting Information

Figure S1 **Myc-PSF2 co-immunoprecipitates Flag-Snm1B in the presence of HA-Mus81.** *A, B,* The indicated Flag epitope-tagged Snm1B (Flag-Snm1B) proteins transiently expressed in 293T cells alone, with Myc epitope-tagged PSF2 (Myc-PSF2), or with Myc-PSF2 and HA epitope-tagged Mus81 (HA-Mus81) were immunoprecipitated (IP) with an anti-Myc antibody and immunoblotted (IB) with an anti-Flag antibody to visualize Flag-Snm1B, anti-Myc antibody to visualize Myc-PSF2, or an anti-Mus81 antibody to visualize Mus81.
(TIF)

Table S1 **Yeast Two-Hybrid Analysis.**
(PDF)
