# Supplementary figures and images for: Snm1B Interacts with PSF2
Source: PLoS One. 2012 Nov 26;7(11):e49626. doi: 10.1371/journal.pone.0049626 (PMC3506659; doi:10.1371/journal.pone.0049626)

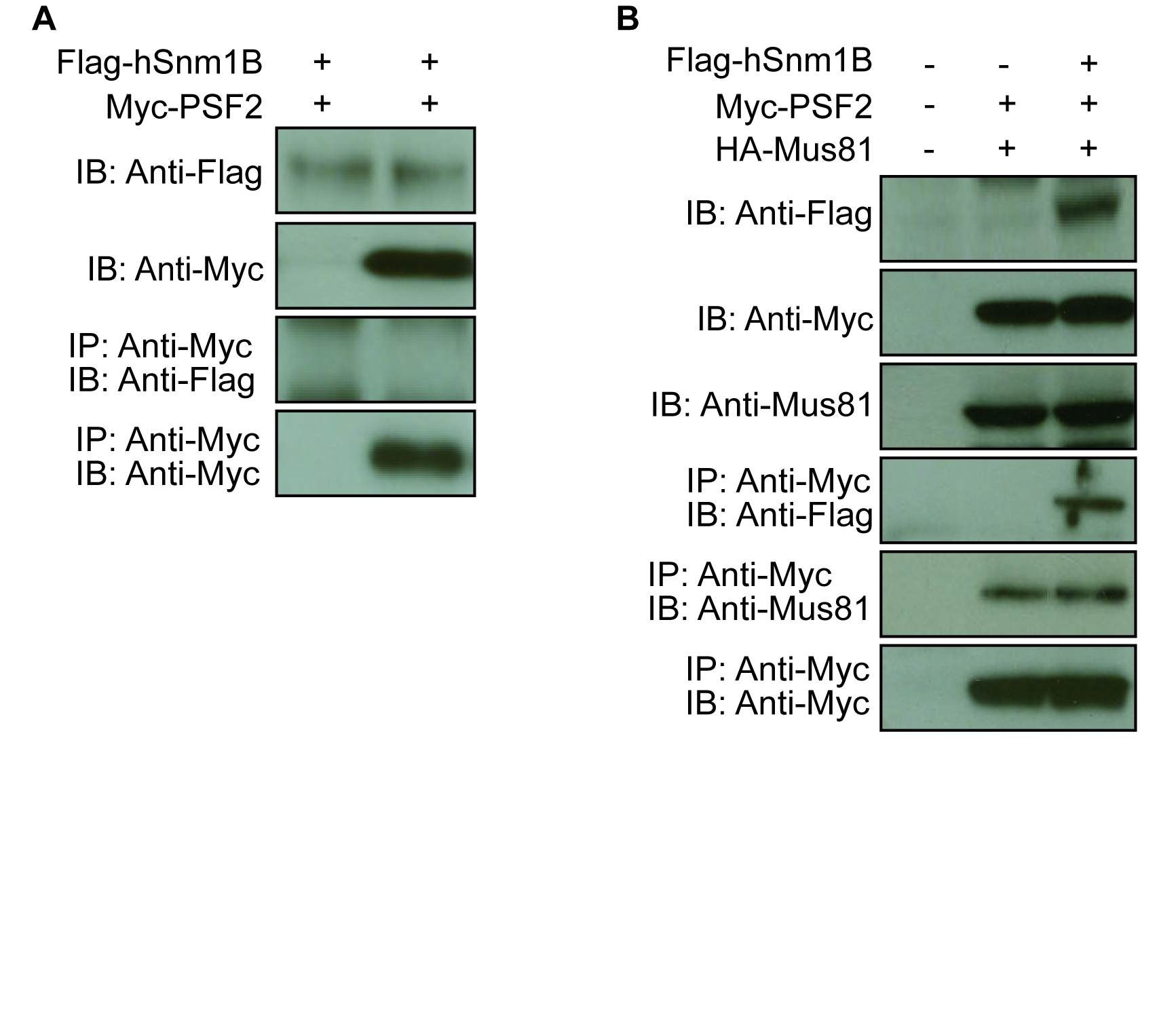

Supplement: Figure S1 — Myc-PSF2 co-immunoprecipitates Flag-Snm1B in the presence of HA-Mus81. A, B, The indicated Flag epitope-tagged Snm1B (Flag-Snm1B) proteins transiently expressed in 293T cells alone, with Myc epitope-tagged PSF2 (Myc-PSF2), or with Myc-PSF2 and HA epitope-tagged Mus81 (HA-Mus81) were immunoprecipitated (IP) with an anti-Myc antibody and immunoblotted (IB) with an anti-Flag antibody to visualize Flag-Snm1B, anti-Myc antibody to visualize Myc-PSF2, or an anti-Mus81 antibody to visualize Mus81. (TIF) [file pone.0049626.s001.tif]
