# Supplementary material for: Snm1B Interacts with PSF2
Source: PLoS One. 2012 Nov 26;7(11):e49626. doi: 10.1371/journal.pone.0049626 (PMC3506659; doi:10.1371/journal.pone.0049626)
Supplement: Table S1 — Yeast Two-Hybrid Analysis. (PDF) [file pone.0049626.s002.pdf]

Table S1. Yeast Two-Hybrid Analysis.

Gene Name	Clones	Sequence
5'-nucleotidase, cytosolic III-like	1	NM_052935.4
adenylate cyclase 9	1	NM_001116.3
aminopeptidase B	1	NM_020216.3
archaelysin family metallopeptidase 2	2	NM_001033574.1
breast cancer associated gene 3	1	NM_020643.2
CD81 molecule	1	NM_004356.3
centrosomal protein 164kDa	1	NM_014956.4
collagen preprotein type4	2	NM_001846.2
collagen type XII	2	NM_080645.2
C-terminal binding protein 2	1	NM_022802.2
cytohesin 3	2	NM_004227.3
ferritin light chain	14	NM_000146.3
G protein-coupled receptor 172A	7	NM_024531.3
guanine nucleotide binding factor beta	5	NM_006098.4
major histocompatibility complex, class I, B	39	NM_005514.6
hypothetical LOC388692	3	NR_027002.1
intestinal alkaline phosphatase precursor	1	NM_001631.3
mesothelin	1	NM_013404.4
microspherule protein 1	3	NM_001012300.1
mitochondrial ribosomal protein L38	1	NM_032478.3
metallothionein 2A	1	NM_005953.3
methylenetetrahydrofolate dehydrogenase 1-like	3	NM_001242768.1
NADH dehydrogenase 1 alpha subcomplex, 3, 9kDa	4	NM_004542.3
pyruvate dehydrogenase complex, component X	1	NM_001135024.1
plectin	2	NM_201384.1
GINS complex subunit 2	3	NM_016095.2
required for meiotic nuclear division 5 homolog B	4	NM_022762.3
ring finger protein 111	3	NM_017610.6
ring finger protein 126	1	NM_194460.2
secretory carrier membrane protein 2	1	NM_005697.3
SWI/SNF related, matrix associated, actin dependent regulator of chromatin	1	NM_003075.3
telomeric repeat binding factor 2	1	NM_005652.3
thioredoxin 2	2	NM_012473.3
unknown	8	
ubiquitin specific peptidase 30	9	NM_032663.3
vitamin K epoxide reductase complex, subunit 1	1	NM_024006.4
Total Sequenced	134	
